# Urothelial cells undergo epithelial-to-mesenchymal transition after exposure to muscle invasive bladder cancer exosomes

**DOI:** 10.1038/oncsis.2015.21

**Published:** 2015-08-17

**Authors:** C A Franzen, R H Blackwell, V Todorovic, K A Greco, K E Foreman, R C Flanigan, P C Kuo, G N Gupta

**Affiliations:** 1 Department of Urology, Loyola University Chicago, Maywood, IL, USA; 2 Department of Pathology, Northwestern Feinberg School of Medicine, Chicago, IL, USA; 3 Oncology Institute, Loyola University Chicago, Maywood, IL, USA; 4 Department of Pathology, Loyola University Chicago, Maywood, IL, USA; 5 Department of Surgery, Loyola University Chicago, Maywood, IL, USA; 6 Department of Radiology, Loyola University Chicago, Maywood, IL, USA

## Abstract

Bladder cancer, the fourth most common noncutaneous malignancy in the United States, is characterized by high recurrence rate, with a subset of these cancers progressing to a deadly muscle invasive form of disease. Exosomes are small secreted vesicles that contain proteins, mRNA and miRNA, thus potentially modulating signaling pathways in recipient cells. Epithelial-to-mesenchymal transition (EMT) is a process by which epithelial cells lose their cell polarity and cell–cell adhesion and gain migratory and invasive properties to become mesenchymal stem cells. EMT has been implicated in the initiation of metastasis for cancer progression. We investigated the ability of bladder cancer-shed exosomes to induce EMT in urothelial cells. Exosomes were isolated by ultracentrifugation from T24 or UMUC3 invasive bladder cancer cell conditioned media or from patient urine or bladder barbotage samples. Exosomes were then added to the urothelial cells and EMT was assessed. Urothelial cells treated with bladder cancer exosomes showed an increased expression in several mesenchymal markers, including α-smooth muscle actin, S100A4 and snail, as compared with phosphate-buffered saline (PBS)-treated cells. Moreover, treatment of urothelial cells with bladder cancer exosomes resulted in decreased expression of epithelial markers E-cadherin and β-catenin, as compared with the control, PBS-treated cells. Bladder cancer exosomes also increased the migration and invasion of urothelial cells, and this was blocked by heparin pretreatment. We further showed that exosomes isolated from patient urine and bladder barbotage samples were able to induce the expression of several mesenchymal markers in recipient urothelial cells. In conclusion, the research presented here represents both a new insight into the role of exosomes in transition of bladder cancer into invasive disease, as well as an introduction to a new platform for exosome research in urothelial cells.

## Introduction

Bladder cancer is the fourth most common noncutaneous malignancy in the United States.^1^ Non-muscle-invasive bladder cancer accounts for approximately 70% of newly diagnosed bladder cancer cases, with the remaining 30% being muscle-invasive bladder cancer (MIBC). Although non-muscle-invasive bladder cancer patients have a high survival rate, the recurrence rate is high, and 10–20% of these patients progress to MIBC.^2^ Although fewer patients are initially diagnosed with MIBCs, they are responsible for the vast majority of bladder cancer-specific deaths.^3, 4^

Exosomes are nanometer-sized microvesicles that are secreted from cells and have important roles in intercellular communication.^5^ Exosomes present various membrane proteins on their surface, allowing them to interact with, and be taken up by, recipient cells. Further, their luminal content includes proteins, mRNA and miRNA.^6, 7, 8^ Prior research has demonstrated that exosomes have a role in cancer biology by promoting survival and growth of disseminated tumor cells; enhancing invasiveness; promoting angiogenesis, migration and tumor cell viability and inhibiting tumor cell apoptosis.^9, 10, 11, 12, 13, 14, 15, 16, 17, 18^

Epithelial-to-mesenchymal transition (EMT) is a biological process in which epithelial cells lose their epithelial characteristics and acquire a migratory, mesenchymal phenotype.^19^ EMT is a complex process that involves cytoskeletal alterations and downregulation of E-cadherin expression.^20^ This loss of epithelial markers and gain of mesenchymal ones has been documented in numerous cancers, including bladder cancer.^21, 22, 23, 24, 25, 26, 27^ During bladder cancer EMT, P-cadherin and N-cadherin expression level increases, followed by the loss of E-cadherin.^28^ In >80% of MIBCs, E-cadherin expression is reduced or completely absent, and there is an upregulation of P-cadherin and/or N-cadherin.^28^ Additionally, mesenchymal markers, twist and vimentin, are associated with bladder cancer stage and grade and may have important roles in bladder cancer progression and metastasis.^24, 29, 30^

In this study, we demonstrate for the first time that exosomes derived from MIBC cells can induce EMT in recipient urothelial cells. We observed increased expression of mesenchymal markers and decreased expression of epithelial markers after urothelial cells were exposed to MIBC exosomes. Further, we found that the MIBC exosomes enhanced the migration and invasion of the urothelial cells, an effect which can be blocked by heparin treatment. Finally, we discovered that exosomes isolated from bladder cancer patient urine or bladder barbotage samples can also induce expression of mesenchymal markers to a similar extent to that induced by MIBC cell line exosomes.

## Results

### MIBC exosomes increase expression of mesenchymal markers in urothelial cells

**Figure 1:**
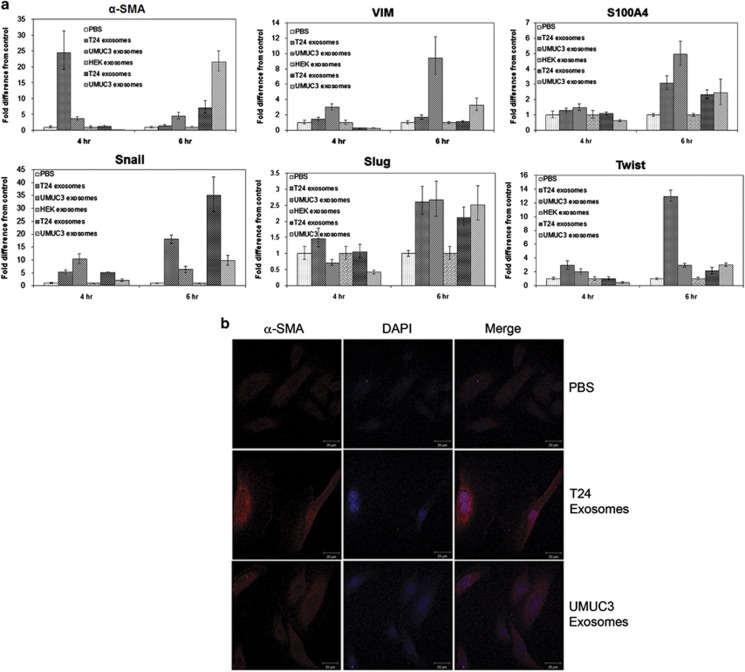
MIBC exosomes increase expression of mesenchymal markers in urothelial cells. (**a**) qRT–PCR for mesenchymal genes expressed in urothelial cells treated with PBS, human embryonic kidney (HEK) exosomes or MIBC exosomes for 4 or 6 h. qRT–PCR was repeated at least three times for each gene. (**b**) Confocal microscopy images of α-SMA expression in urothelial cells treated with PBS or MIBC exosomes for 24 h. Confocal microscopy was performed at least three times. A representative image is shown. Scale bar=20 μm. DAPI, 4,6-diamidino-2-phenylindole.

Previous studies have demonstrated that an increase in the expression of mesenchymal markers, such as vimentin, snail and twist, are associated with increased bladder cancer grade and stage.^24, 30^ Moreover, studies have shown that bladder cancer cell lines shed exosomes containing proteins important for tumor progression.^9, 10, 11^ Therefore, we wanted to determine whether exosomes shed from invasive bladder cancer cell lines could induce the expression of mesenchymal markers in primary urothelial cells. To test this, we isolated exosomes from two MIBC cell lines using previously established methods.^31^ We then treated primary urothelial cells with the exosomes for 4 or 6 h, isolated mRNA and performed quantitative reverse transcriptase–PCR (qRT–PCR) to evaluate the expression of several mesenchymal genes. The 4- and 6-h time points were chosen to help differentiate between mRNAs originating from the MIBC exosomes and newly transcribed mRNAs synthesized in response to exosome-associated transcription factors. We observed an increase in several mRNAs for mesenchymal markers, including α-smooth muscle actin (α-SMA), vimentin, S100A4, snail and twist, when primary urothelial cells were treated with exosomes isolated from invasive bladder cancer cell lines, as compared with the phosphate-buffered saline (PBS)-treated cells or to cells treated with exosomes isolated from human embryonic kidney cells, a non-transformed normal cell line (Figure 1a). Interestingly, gene expression varied between the exosomes derived from the two cell lines, suggesting that, even though T24 and UMUC3 are both muscle-invasive transitional cell carcinoma cell lines, their exosomes likely contain different genetic cargo.

As α-SMA mRNA expression was elevated in the primary urothelial cells at both 4 and 6 h following exposure to either T24- or UMUC3-derived exosomes, we wanted to determine whether there was also an increase in α-SMA protein expression. The urothelial cells were treated with MIBC exosomes for 24 h, then fixed and immunostained for α-SMA. We observed an increase in α-SMA expression in the cells treated with T24- or UMUC3-derived exosomes, as compared with control (PBS)-treated cells (Figure 1b).

### MIBC exosomes decrease expression and alter localization of E-cadherin and β-catenin in urothelial cells

**Figure 2:**
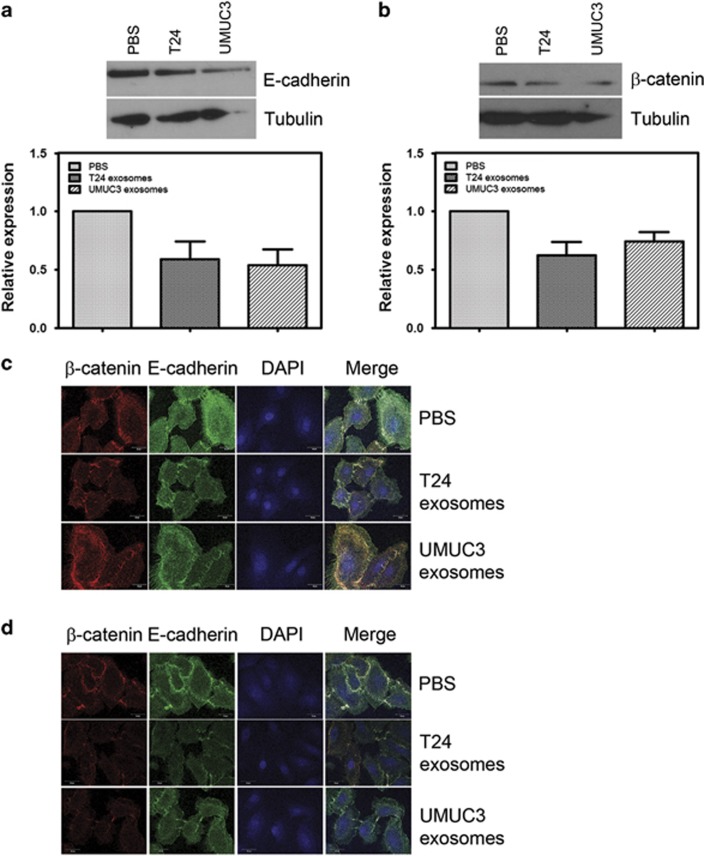
MIBC exosomes decrease expression and alter localization of E-cadherin and β-catenin in urothelial cells. (**a**) Representative western blotting of E-cadherin expression in urothelial cells after 48 h of treatment with PBS or MIBC exosomes. The bar graph shows the quantitation of E-cadherin expression from four experiments. (**b**) Representative western blotting of β-catenin expression in urothelial cells after 48 h of treatment with PBS or MIBC exosomes. The bar graph shows the quantitation of β-catenin expression from four experiments. (**c**, **d**). Confocal microscopy images of β-catenin and E-cadherin expression and localization in urothelial cells treated with PBS or MIBC exosomes for (**c**) 24 or (**d**) 48 h. Confocal microscopy was performed at least three times. A representative image is shown. Scale bar=20 μm. DAPI, 4,6-diamidino-2-phenylindole.

Previous studies have shown that decreased expression of epithelial markers E-cadherin and β-catenin is associated with bladder cancer progression,^24, 30^ and E-cadherin is reduced or absent in >80% of MIBCs.^28^ Thus we wanted to determine whether E-cadherin and β-catenin were downregulated in the primary urothelial cells treated with MIBC exosomes. Protein expression of E-cadherin and β-catenin after 48 h treatment with MIBC exosomes was downregulated in the primary urothelial cells (Figures 2a and b). We also observed a decrease in E-cadherin and β-catenin expression, as well as a change in their localization, by confocal microscopy (Figures 2c and d).

In epithelial cells, when E-cadherin interacts with β-catenin, it becomes anchored to the actin cytoskeleton where it provides mechanical stability to the cell-to-cell junctions.^32^ When E-cadherin is downregulated, β-catenin is released and translocates to the nucleus to activate WNT signaling, leading to EMT and metastasis.^33^ As expected, in the PBS-treated primary urothelial cells, E-cadherin and β-catenin co-localized at cell–cell junctions (Figures 2c and d). Upon treatment with MIBC exosomes, we found some weak staining of E-cadherin at the cell borders. However, β-catenin was almost completely absent from the cell borders. In addition, we detected β-catenin in the nucleus of the 24 h MIBC exosome-treated urothelial cells (Figure 2c).

### MIBC exosomes alter motility in primary urothelial cells

**Figure 3:**
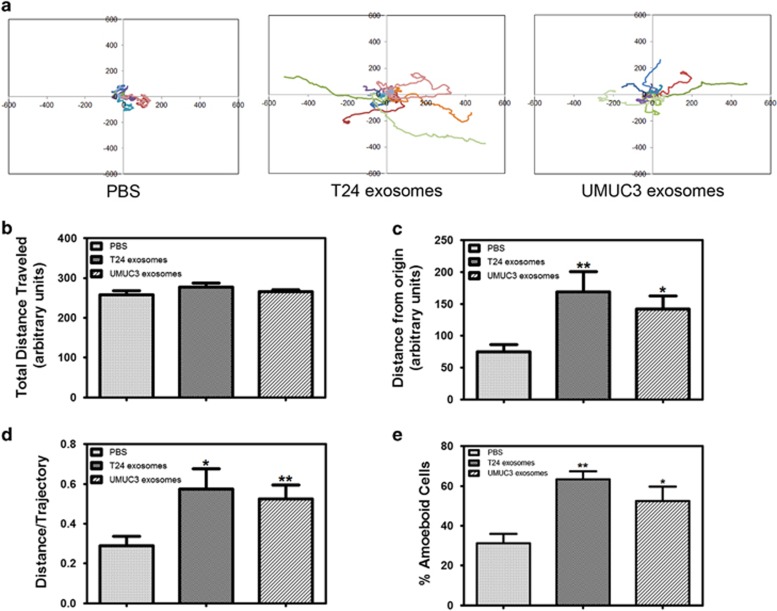
MIBC exosomes alter motility in primary urothelial cells. Urothelial cells were plated on Col IV-coated cover glass with PBS or MIBC exosomes and allowed to attach for 1 h before being placed in the ASMDW for live cell imaging. (**a**) Wind-rose plots of cell tracks from time-lapse microscopy. Each wind-rose plot shows centroid tracks from 10 representative tracks, with the initial position of each track superimposed at 0,0 for clarity. (**b**) Total distance traveled over 6 h was calculated using the ImageJ imaging software. (**c**) The average distance from the origin over 6 h for cells treated with MIBC exosomes versus control (40 cells/condition were analyzed). (**d**) The distance/trajectory was calculated as a ratio of the distance from the origin traveled over the total distance traveled. (**e**) The percentage of cells undergoing amoeboid cell motility was calculated as the number of cells displaying amoeboid-type movement divided by the total number of cells for each treatment. Data are represented as average±s.e.m. *Difference between control and MIBC exosome-treated cells (*P*<0.05) by a paired Student's *t*-test. **Difference between control and MIBC exosome-treated cells (*P*<0.01) by a paired Student's *t*-test.

EMT has been shown to be associated with cancer cell invasion and metastasis in several malignancies, including bladder cancer.^27^ We found that β-catenin is being translocated to the nucleus in MIBC exosome-treated epithelial cells (Figures 2c and d), which can lead to EMT and metastasis.^33^ Therefore, we wanted to determine whether MIBC exosomes can influence the motility of the primary urothelial cells. We plated the urothelial cells in the presence of MIBC exosomes (or PBS) on the bladder epithelial basement membrane component, Collagen IV (Col IV)-coated chamber glass.^34^ Cells were allowed to attach for 1 h, and then live cell imaging was performed (Supplementary Videos S1–S3). Figure 3a shows wind-rose plots for 10 individual tracks per treatment. Although the total distance traveled was fairly similar in all treatments (Figure 3b), the T24- and UMUC3-treated cells moved farther from the origin, and they had an increased distance/trajectory (or cell persistence) (Figures 3c and d). We also observed that the MIBC exosome-treated cells exhibited increased amoeboid migration as compared with the control-treated cells (Figure 3e). Cells that exhibit amoeboid-like movement have enhanced contractility, which allows them to squeeze through gaps in the extracellular matrix fibers.^35, 36, 37^

### MIBC exosomes enhance the migration and invasion of primary urothelial cells

**Figure 4:**
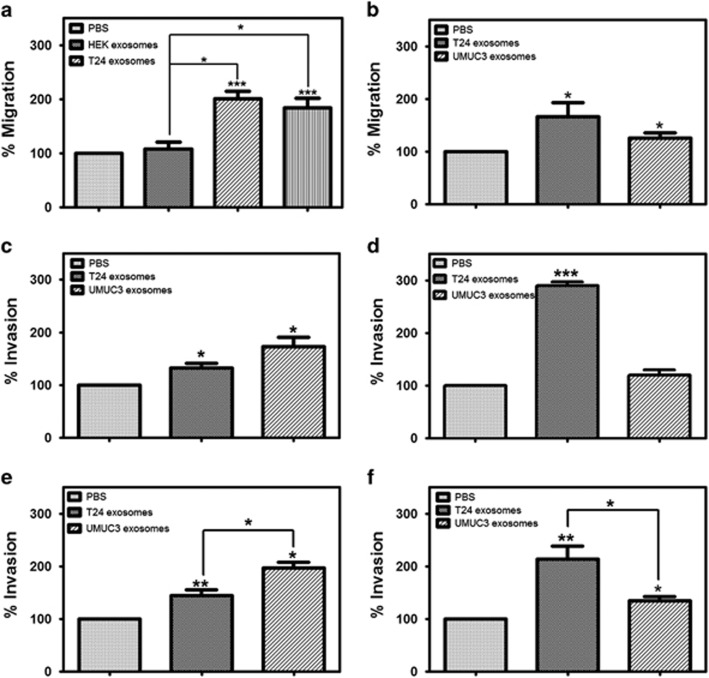
MIBC exosomes enhance the migration and invasion of primary urothelial cells. (**a**) Urothelial cells were plated on uncoated transwell inserts in 500 μl serum-free media with MIBC exosomes, human embryonic kidney (HEK) exosomes or PBS for a migration assay. (**b**) Urothelial cells were plated on uncoated transwell inserts in 500 μl serum-free media, and MIBC exosomes (30 μg/ml) or PBS were plated in the bottom chamber. (**c**) Urothelial cells were plated on Col IV-coated transwell inserts in 500 μl serum-free media with MIBC exosomes (30 μg/ml) or PBS for an invasion assay. (**d**) Urothelial cells were plated on Col IV-coated transwell inserts in 500 μl serum-free media, and MIBC exosomes (30 μg/ml) or PBS were plated in the bottom chamber. (**e**) Urothelial cells were plated on matrigel-coated transwell inserts in 500 μl serum-free media with MIBC exosomes (30 μg/ml) or PBS for an invasion assay. (**f**) Urothelial cells were plated on matrigel-coated transwell inserts in 500 μl serum-free media, and MIBC exosomes (30 μg/ml) or PBS were plated in the bottom chamber. Migration and assays were carried out in triplicate. Graphs represent averages (normalized to the PBS control-treated cells)±s.e.m. *P<0.05, **P<0.01, ***P<0.001, by a paired Student's *t*-test.

Expanding on our findings from the live cell imaging studies, we next examined the effect of MIBC-derived exosomes on primary urothelial cell migration and invasion in a transwell system. To test the effect of MIBC exosomes on the urothelial cell migration, cells were plated in the presence of MIBC exosomes (T24 or UMUC3) in the top chamber of a transwell system. The cells were allowed to migrate for 24 h before being fixed and stained. Both T24 and UMUC3 exosomes enhanced the migration of the primary urothelial cells (Figure 4a). Treating the primary urothelial cells with exosomes derived from human embryonic kidney cells did not enhance migration (Figure 4a), demonstrating that it is not just the presence of exosomes but specifically MIBC exosomes that is responsible for the augmentation of cell migration. Similar results were obtained when primary urothelial cells were treated with exosomes derived from primary human fibroblast cells (data not shown). Several studies have demonstrated that cancer exosomes can serve as chemoattractants for cell migration and invasion.^20, 38, 39^ Thus we next sought to determine whether the T24 or UMUC3 exosomes could serve as a chemoattractant for cell migration. For these studies, we plated the primary urothelial cells in the top transwell chamber and the exosomes were added to serum-free media in the bottom chamber. Interestingly, while UMUC3 exosomes had a modest effect on urothelial cell migration, T24 exosomes showed a more pronounced effect (Figure 4b).

We next wanted to determine whether the MIBC exosomes could enhance the invasion of the urothelial cells. The cells were plated in the presence of MIBC exosomes (T24 or UMUC3) on Col IV or matrigel in the top chamber of a transwell system. The cells were allowed to invade for 24 h before being fixed and stained. Although exosomes from both MIBC cell lines enhanced invasion through both Col IV and matrigel, we observed greater invasion in the cells treated with the UMUC3-derived exosomes (Figures 4c and e). However, when the MIBC exosomes were added to the bottom chamber of the transwell system, UMUC3-derived exosomes had only a slight effect on invasion, while the T24-derived exosomes significantly enhanced the invasion of the urothelial cells (Figures 4d and f).

### Heparin blocks the effect of MIBC exosomes on cell migration and invasion

**Figure 5:**
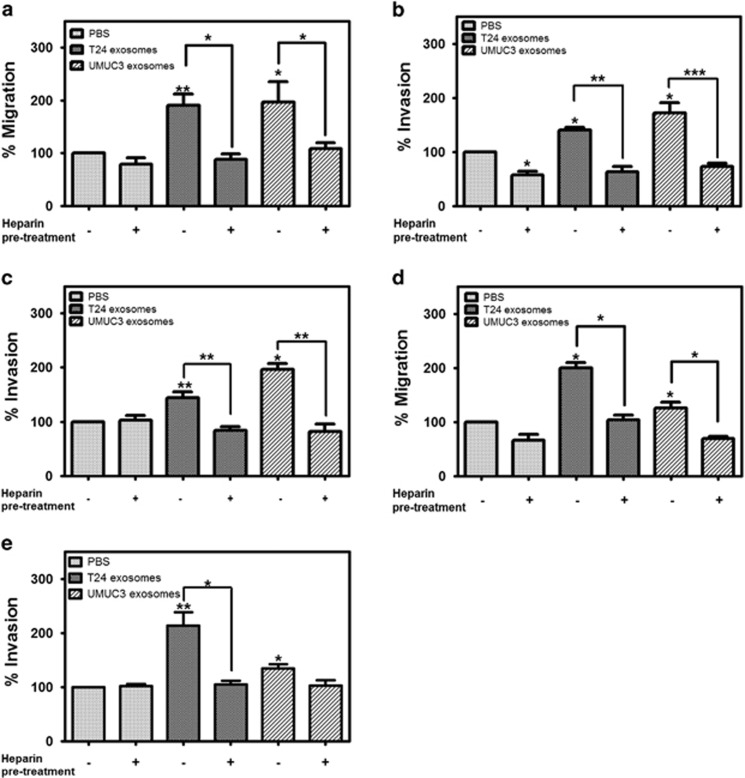
Heparin blocks the effect of MIBC exosomes on cell migration and invasion. (**a**–**c**) Urothelial cells were pretreated with heparin (10 μg/ml) for 30 min and then plated for migration on uncoated transwell inserts (**a**), Col IV-coated inserts (**b**) or matrigel-coated inserts (**c**) in 500 μl serum-free media with MIBC exosomes or PBS for migration or invasion assays. (**d**, **e**) MIBC exosomes were pretreated with heparin for 30 min. Urothelial cells were plated on uncoated transwell inserts (**d**) or matrigel-coated inserts (**e**) in 500 μl serum-free media, and MIBC exosomes (30 μg/ml) or PBS were plated in the bottom chamber. Migration and invasion assays were carried out in triplicate. Graphs represent averages (normalized to the PBS control-treated cells)±s.e.m. *P<0.05, **P<0.01, ***P<0.001, by a paired Student's *t*-test.

Our previous study demonstrated that pretreatment of bladder cells with heparin partially blocked exosome uptake, suggesting that uptake occurs through a heparin sulfate proteoglycan (HSPG)-dependent mechanism.^31^ Furthermore, it has been shown that heparin treatment can attenuate exosome-dependent cell migration in glioblastoma and CHO cell lines.^40^ Therefore, we decided to test whether pretreating the urothelial cells with heparin would blunt the effect of MIBC exosomes on migration and invasion. We pretreated primary urothelial cells with heparin prior to plating them in the presence of MIBC exosomes in the top chamber of a transwell migration system. Heparin pretreatment reduced the effect of MIBC exosomes on cell migration compared with the PBS control (Figure 5a). When the primary urothelial cells were pretreated with heparin prior to being plated in the presence of MIBC exosomes (or PBS control) on Col IV-coated inserts, decreased invasion was observed in all conditions (Figure 5b), suggesting that heparin was interfering with the cells' ability to interact with Col IV (as Col IV is a ligand for HSPGs). When the primary urothelial cells were pretreated with heparin prior to being plated in the presence of MIBC exosomes (or PBS control) on matrigel-coated inserts, heparin completely ablated the effect of the MIBC exosomes on invasion (Figure 5c). Remarkably, when the MIBC exosomes were pretreated with heparin and then placed in the bottom chamber of the transwell system, they were no longer able to enhance the migration or matrigel invasion of the primary urothelial cells (Figures 5d and e).

### Exosomes isolated from bladder cancer patient urine and barbotage samples increase expression of mesenchymal markers in and enhance the migration of primary urothelial cells

**Figure 6:**
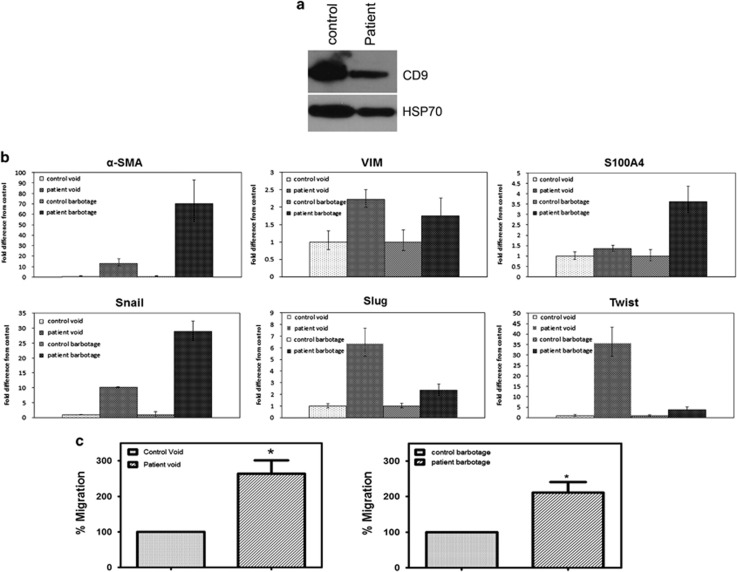
Exosomes isolated from bladder cancer patient urine and barbotage samples increase expression of mesenchymal markers in and enhance the migration of primary urothelial cells. (**a**) Western blotting demonstrating that exosomal markers are expressed in the exosomes isolated from control and bladder cancer urinary exosomes. (**b**) qRT–PCR for mesenchymal genes expressed in urothelial cells treated with control or patient urinary or barbotage exosomes for 4 h. qRT–PCR was repeated in triplicate three times for each gene. (**c**) Urothelial cells were plated on uncoated transwell inserts in 500 μl serum-free media with control urinary exosomes or bladder cancer patient urinary exosomes for a migration assay. (**d**) Urothelial cells were plated on uncoated transwell inserts in 500 μl serum free media with control barbotage exosomes or bladder cancer patient barbotage exosomes for a migration assay. Migration assays were carried out in triplicate. Graphs represent averages (normalized to the control-treated cells)±s.e.m. *P<0.05, by a paired Student's *t*-test.

We next sought to determine whether the changes we observed could be replicated with patient samples. Urine and bladder barbotage samples were collected from bladder cancer patients and patients without cancer who were undergoing cytoscopic procedures. Exosomes were isolated from the urine and bladder barbotage samples and a western blotting was performed to confirm that we had isolated urinary exosomes (Figure 6a). We then treated the primary urothelial cells with the urinary or barbotage exosomes and collected RNA samples at 4 h. Similarly to what we saw with the MIBC cell exosomes, we observed an increase in mesenchymal markers in cells treated with exosomes from bladder cancer patients, as compared with the controls (Figure 6b). Although we were able to detect significant increases in mesenchymal genes in patient urine samples as compared with control urine samples, we saw a more pronounced increase in the expression of mesenchymal markers in the barbotage samples compared with the barbotage controls. We wanted to demonstrate that the exosomes isolated from the patient samples could affect cell migration. To test the effect of bladder cancer patient exosomes on urothelial cell migration, cells were plated in the presence of control or bladder cancer patient exosomes in the top chamber of a transwell system. The cells were allowed to migrate for 24 h before being fixed and stained. Exosomes isolated from bladder cancer patient samples (urine or barbotage samples) enhanced the migration of the primary urothelial cells, as compared with their respective controls (Figures 6c and d).

## Discussion

EMT has been shown to be implicated in the pathogenesis of bladder cancer,^24, 25, 26, 27^ and exosomes are known to have a role in tumor progression.^9, 10, 11, 12, 13, 14, 15^ Understanding the role that exosomes have in EMT in bladder cancer can provide insight into the mechanisms of bladder cancer progression and recurrence, which are currently lacking.

To our knowledge, this is the first study examining the ability of cancer exosomes to induce EMT. In this study, treatment of urothelial cells with MIBC exosomes led to increased expression of mesenchymal genes and decreased expression of E-cadherin and β-catenin. Further studies are warranted to determine the mechanism by which MIBC exosomes are regulating these proteins. Increased migration and invasion of urothelial cells treated with the MIBC exosomes was also observed. Our live cell imaging studies provide insight into how the exosomes are altering cell motility. We established that urothelial cells treated with MIBC exosomes move more persistently and further from the origin than the control cells. It would be interesting to determine whether we see the same pattern of cell motility when the cells are plated on other extracellular matrix proteins, such as major basement membrane component laminin 332.

We observed that there was differential expression of mesenchymal genes in the urothelial cells treated with T24 or UMUC3 cell exosomes. We further discovered that T24 and UMUC3 exosomes can exert distinctive effects on urothelial cell migration and invasion. This suggests that there are most likely differences in the cargo of T24 and UMUC3 exosomes. In order to gain some insight into potential differences in exosomal cargo, we determined the expression levels of the mesenchymal genes in the T24 and UMUC3 exosomes. The expression of vimentin, a gene that is one of the main drivers of EMT and motility,^27^ was significantly higher in the UMUC3 exosomes, as compared with the T24 exosomes (Supplementary Figure 1). It is tempting to speculate on differences in the levels of other relevant proteins and RNA transferred, as we observed a greater enhancement of urothelial cell migration and invasion when the cells were treated with UMUC3 exosomes. In addition, T24 exosomes exert the chemoattractant ability seemingly independently of their ability to directly deliver cargo to the target cells (Figure 4). This ability, which has been observed before,^39, 40^ brings up the possibility of a different method of delivery by exosomes through a heretofore unknown mechanism.

Our live cell imaging studies demonstrate that urothelial cells treated with MIBC exosomes move further from the origin and more persistently. We also observed an increased number of exosome-treated cells exhibiting amoeboid cell movement, as compared with the control cells. Cells that utilize amoeboid migration can move in 3D substrates independently of extracellular matrix degradation.^37, 41^ As expected, we also observed increased invasion in the MIBC-treated cells, suggesting that amoeboid migration may be contributing to the increased invasion. Future studies are warranted to determine whether MIBC are activating RhoA/ROCK signaling pathway, which has been shown to promote the enhanced contractility of cells using amoeboid migration.^41, 42^

Our data suggest that the effects of the MIBC exosomes on migration and invasion are mediated via HSPGs. We previously demonstrated that HSPGs are, at least partially, responsible for exosome uptake. As HSPGs often act as co-receptors for various integrin receptors,^43, 44, 45^ and integrin receptors are well-established mediators of cell–matrix interactions (including tumor migration and invasion),^46, 47, 48^ we would hypothesize that integrin receptors are involved in the enhancement of urothelial migration and invasion by MIBC exosomes. We previously found that pretreating exosomes with heparin had a minimal effect on their uptake. However, in this study, pretreating the exosomes with heparin completely abrogated the chemoattractant effect of the exosomes on cell migration and invasion. This suggests that the exosomes may be stimulating migration through an HSPG-dependent mechanism. A study by Christianson *et al.*^40^ also found that blocking HSPGs attenuated the effects of exosomes on cell migration in glioblastoma cells; however, the mechanism of this inhibition remains to be elucidated.

We demonstrated that voided urinary and bladder barbotage sample exosomes isolated from bladder cancer patients were able to induce the expression of mesenchymal genes in recipient urothelial cells. It is intriguing that we see a more pronounced effect with the barbotage exosomes than with the urinary exosomes. In the bladder barbotage, the bladder is rinsed with saline, which most likely results in the extrication of newly shed vesicles, as well as vesicles that may be loosely interacting with target cells. Voided urine samples, on the contrary, contain exosomes originating throughout the urinary tract (for example, kidney, ureter, prostate). Thus we may be effectively enriching the population of bladder cancer exosomes in barbotage samples, which can account for the increased induction of mesenchymal markers in the recipient urothelial cells.

Taken together, our data provide a first glimpse into the role of exosomes in bladder cancer progression, as well as recurrence. This provides a platform for elucidating the mechanism in which exosomes are inducing EMT in urothelial cells, as well as insight into determining predictive biomarkers.

## Materials and methods

### Cell culture

UMUC3 human bladder cancer cell line was purchased from ATCC (Manassas, VA, USA) and cultured in Dulbecco's modified Eagle's medium containing 10% fetal bovine serum, 100 units/ml penicillin, 100 μg/ml streptomycin and 2 mmol/l L-glutamine. T24 human bladder cancer cell line was purchased from ATCC and cultured in McCoys media containing 10% fetal bovine serum, 100 units/ml penicillin, 100 μg/ml streptomycin and 2 mmol/l L-glutamine. Primary urothelial cells were purchased from Zen-Bio (Research Triangle Park, NC, USA) and cultured in CnT-Prime Epithelial Culture Medium. Human embryonic kidney cells were from ATCC and cultured in Dulbecco's modified Eagle's medium containing 10% fetal bovine serum, 100 units/ml penicillin, 100 μg/mL streptomycin and 2 mmol/l L-glutamine.

### Reagents and antibodies

QuantiTect primer assays for α-SMA, vimentin, S100A4, Snail, Slug, Twist and glyceraldehyde 3-phosphate dehydrogenase were from Qiagen (Valencia, CA, USA). E-cadherin monoclonal antibody for immunofluorescence studies), β-catenin polyclonal antibodies (for immunofluorescence studies) and α-SMA polyclonal antibodies were from AbCam (Cambridge, MA, USA). Alexa 488-conjugated goat anti-mouse and Alexa 594-conjugated goat anti-rabbit secondary antibodies were from Life Technologies (Carlsbad, CA, USA). E-cadherin monoclonal antibody (for immunoblotting studies), β-catenin monoclonal antibody (for immunoblotting studies) and horseradish peroxidase-conjugated goat anti-rabbit secondary antibodies were from Cell Signaling Technologies (Danvers, MA, USA). Horseradish peroxidase-linked goat anti-mouse secondary antibodies were from GE Healthcare (Pittsburgh, PA, USA). Col IV was from BD Biosciences (San Jose, CA, USA). Uncoated and Matrigel-coated Transwell plates and heparin sodium salt were from Fisher Scientific (Waltham, MA, USA).

### Exosome isolation from bladder cancer cell lines and urine/barbotage samples

Exosomes were isolated from MIBC cell conditioned media, voided urine or bladder barbotage samples by differential centrifugation, as previously described.^49^ Conditioned media, urine or barbotage samples were centrifuged at 300 *g* for 10 min to remove contaminating cells. The supernatant was collected and centrifuged at 2000 *g* for 10 min to pellet dead cells. The supernatant was then ultracentrifuged at 100 000 *g* for 70 min. The pellets were washed with PBS, pooled and ultracentrifuged at 100 000 *g* for 70 min. The final pellet was resuspended in PBS or RIPA buffer or RNA lysis buffer. Exosome protein concentration was determined using the BCA protein assay (Fisher Scientific), and 30 μg/ml of the exosome suspension was used for all experiments. The collection of voided urine and barbotage samples was approved by the Institutional Review Board at Loyola University Chicago.

### Quantitative reverse transcriptase–PCR

RNA was isolated and purified from primary urothelial cells treated with PBS, T24-derived exosomes or UMUC3-derived exosomes using the miRVana mRNA Isolation Kit (Life Technologies) according to the manufacturer's recommendations. First-strand synthesis was performed on normalized RNA samples using iScript Reverse Transcription Supermix (Bio-Rad, Philadelphia, PA, USA). qRT–PCR was performed using SYBR Green Super Mix (Bio-Rad) with QuantiTect primer assays for α-SMA, vimentin, S100A4, snail, slug, twist and glyceraldehyde 3-phosphate dehydrogenase. qRT–PCR was performed in triplicate three times for each gene. Data are shown as mean±s.d. Data using patient samples show an average of bladder cancer patient exosome-treated urothelial cells (*n*=6) to control exosome-treated urothelial cells (*n*=4).

### Time-lapse motility assays

Primary urothelial cells were detached and plated on Collagen IV-coated four-well chambered cover glass in media containing PBS or exosomes derived from T24 or UMUC3 bladder cancer cell lines. Cells were allowed to attach for 60 min, and then they were placed in the ASMDW for 6 h, with phase time-lapse recordings taken at 2-min intervals using a × 20 objective. Quantitations were made using the ImageJ imaging software (Bethesda, MD, USA). Data are shown as mean±s.e.m. Statistics were performed using a paired Student's *t*-test.

### Immunofluorescence

Primary urothelial cells were grown on glass coverlsips. The cells were treated with T24- or UMUC3-derived exosomes (or PBS control) for 24 or 48 h. The coverslips were washed with PBS, fixed with 4% paraformaldehyde and permeabilized with 0.1% Triton X-100. They were then incubated with mouse anti-E-cadherin (1:100) and rabbit β-catenin (1:100) antibodies or rabbit α-SMA (1:100) antibodies for 1 h at 37 °C. Primary antibodies were visualized using Alexa Fluor 488- and 594-conjugated goat secondary antibodies against mouse and rabbit (1:1000). Coverslips were examined using a Zeiss LSM-510 confocal microscope (Thornwood, NY, USA) and LSM imaging software (Thornwood, NY, USA). Immunofluorescence was performed in duplicate three times for each treatment.

### Immunoblotting

Primary urothelial cells were plated in six-well plates and allowed to attach and spread. Cells were treated with PBS or exosomes derived from T24 or UMUC3 cells and harvested 24 and 48 h later with RIPA buffer and subjected to sodium dodecyl sulfate–polyacrylamide gel electrophoresis. After transfer to a polyvinylidene difluoride membrane, immunoblot analysis was performed with antibodies against E-cadherin, β-catenin and tubulin. Quantitations of band intensities were carried out using Analyze Gels tool of the ImageJ software. Quantitation of band intensities was performed from four separate experiments.

### Migration and invasion assays

The transwell migration assay and the Col IV and matrigel invasion assays were performed as previously described^50^ with the following modifications. For the migration assays, cells were detached using accutase cell detachment solution (Zen-bio, Research Triangle Park, NC, USA) and washed with serum-free medium, and 5 × 10^4^ cells were added to the transwell inserts (0.8 μm; BD Biosciences) in a volume of 500 μl with MIBC exosomes (30 μg/ml) or PBS. The outer wells contained 750 μl of basal medium. Cells were allowed to migrate for 24 h; non-migrating cells were removed from inner wells using a cotton swab, and migrating cells adherent to the bottom of the membrane were fixed and stained using a Diff-Quick Staining Kit (Fisher Scientific). Migrating cells were enumerated by upright stereo microscope using a × 10 objective (Nikon, Melville, NY, USA). Assays were performed four times in triplicate. Data are shown as mean±s.e.m. Statistics were performed using a paired Student's *t*-test.

For Collagen IV invasion, type IV collagen was dissolved in 0.5 M HCl at a concentration of 10 μg/ml. The transwell inserts (0.8 μm; BD Biosciences) were coated with collagen overnight at 4 °C. Collagen-coated inserts were then washed with basal media to remove salts and used immediately. Cells were detached and plated as described above. Assays were performed four times in triplicate. Data are shown as mean±s.e.m. Statistics were performed using a paired Student's *t*-test.

For the matrigel invasion assay, precoated plates were used. The matrigel-coated inserts were rehydrated prior to use, according to the manufacturer's instructions. Cells were detached and plated as described above. Assays were performed four times in triplicate. Data are shown as mean±s.e.m. Statistics were performed using a paired Student's *t*-test.
