# Supplementary figures and images for: Urothelial cells undergo epithelial-to-mesenchymal transition after exposure to muscle invasive bladder cancer exosomes
Source: Oncogenesis. 2015 Aug 17;4(8):e163–. doi: 10.1038/oncsis.2015.21 (PMC4632072; doi:10.1038/oncsis.2015.21)

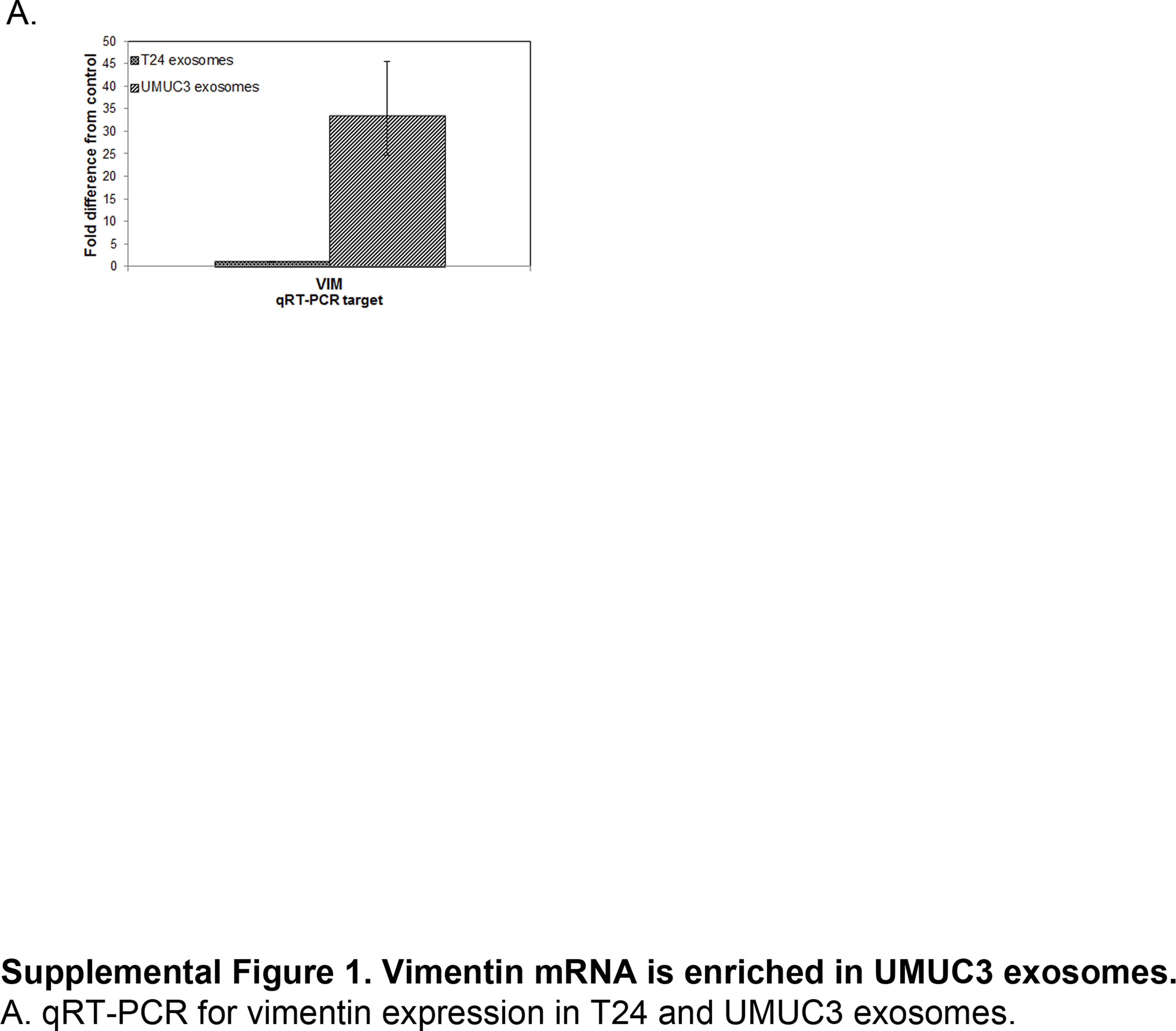

Supplement: Supplementary Figure 1 [file oncsis201521x1.tif]
